# Effects of Hand Proximity and Movement Direction in Spatial and Temporal Gap Discrimination

**DOI:** 10.3389/fpsyg.2016.01930

**Published:** 2016-12-09

**Authors:** Michael Wiemers, Martin H. Fischer

**Affiliations:** ^1^Division of Cognitive Science, University of PotsdamPotsdam, Germany; ^2^Donders Institute for Brain, Cognition and Behaviour, Radboud UniversityNijmegen, Netherlands

**Keywords:** attention, perception and action, two visual systems, visual perception, movement preparation

## Abstract

Previous research on the interplay between static manual postures and visual attention revealed enhanced visual selection near the hands (near-hand effect). During active movements there is also superior visual performance when moving toward compared to away from the stimulus (direction effect). The “modulated visual pathways” hypothesis argues that differential involvement of magno- and parvocellular visual processing streams causes the near-hand effect. The key finding supporting this hypothesis is an increase in temporal and a reduction in spatial processing in near-hand space (Gozli et al., 2012). Since this hypothesis has, so far, only been tested with static hand postures, we provide a conceptual replication of Gozli et al.’s (2012) result with moving hands, thus also probing the generality of the direction effect. Participants performed temporal or spatial gap discriminations while their right hand was moving below the display. In contrast to Gozli et al. (2012), temporal gap discrimination was superior at intermediate and not near hand proximity. In spatial gap discrimination, a direction effect without hand proximity effect suggests that pragmatic attentional maps overshadowed temporal/spatial processing biases for far/near-hand space.

## Introduction

It is a widely held assumption that our ability to selectively direct attention in space originates from a specialized fronto-parietal network which is independent from the perceptuo-motor system (cf. Posner and Dehaene, 1994). In direct contrast to this idea, the premotor theory of attention argues that spatial attention depends on activation in the motor system, in the sense that shifts of attention are generated by the preparation of eye- or reaching movements (Rizzolatti et al., 1987). This view proposes “pragmatic maps of attention” that reflect one’s current action tendencies (Rizzolatti et al., 1994). Considerable evidence supports the pragmatic maps proposal: Initial support came from the meridian effect which reflects delayed visual detection when a target is presented in the opposite compared to the same visual hemifield as a previously presented cue (Hoffman and Subramaniam, 1995; Deubel and Schneider, 1996). This observation suggests that attentional shifts depend on updating of the direction parameter of planned eye movements. Also in line with the pragmatic maps proposal, coupling of motor preparation and attentional selection has been observed for reaching movements (Tipper et al., 1992; Fischer, 1997; Deubel et al., 1998; Baldauf and Deubel, 2008). For example, Tipper et al. (1992) observed that in a reaching task distractors within the path between the hand and the target cause interference, whereas distractors located behind the target do not, suggesting that attention is guided by action-centered representations. Similarly, Fischer (1997, Experiment 1) found enhanced letter discrimination in the visual hemifield to which the participant’s hand was moving, indicating that attention was guided by movement-related processing. More recent studies confirm that, during reaching movements, visual attention is tightly coupled to action-relevant locations (e.g., Baldauf and Deubel, 2008). These findings indicate that motor preparation generally drives visual selection toward intended goal locations *ahead* of the current position of the hand, leading to perceptual advantages.

Interestingly, in addition to the coupling between active motor preparation and attentional selection, visual selection processes appear to be altered by the mere proximity between the static hands and the stimulus (for a recent review see Goodhew et al., 2015). One of the first studies in this context tested the influence of hand-stimulus proximity in a standard covert attentional orienting paradigm (Reed et al., 2006). A lateralized visual target was preceded by a peripheral cue which was valid in 70% of trials. Hand proximity was manipulated by placing one hand on the left or right side of the screen. Consequently, stimuli on the same side as the hand were in near-hand space whereas stimuli on the opposite side as the hand were in far-hand space. Overall, responses were facilitated for stimuli presented in near-hand space, which the authors interpreted as evidence for attentional prioritization of near-hand space. This attentional account of hand proximity effects was qualified by Abrams et al. (2008), who investigated the effect of hand proximity for three classical attentional paradigms. Abrams et al. (2008) found that visual search times were slowed when holding the display between one’s hands, which the authors interpreted as a delay of attentional disengagement mechanisms due to detailed processing of near-hand space. In line with this hypothesis, the inhibition to reengage attention onto a previously attended location (inhibition of return effect) was reduced. Moreover, they observed an increase of the attentional blink, that is, impaired target identification shortly after a previous target in a rapid serial visual presentation task. The attentional blink is believed to reflect disengagement processes before attention can engage a new stimulus. Thus, attentional prioritization of the hands seems to induce a perceptual disadvantage.

While these and other recent studies on the influence of hand proximity on visual selection (Davoli and Brockmole, 2012; Kelly and Brockmole, 2014; Le Bigot and Grosjean, 2016) have started to provide new insights into the role of the motor system in attention deployment, these experimental manipulations have generally been restricted to the use of static hand positions. Facilitatory effects of motor planning and inhibitory effects of near-hand space seem to indicate a competition between different mechanisms for perception-action coupling. This methodological difference between static and dynamic approaches to the relationship between visual attention and action also limits our knowledge about attention deployment in more realistic tasks, such as manipulating hand-held devices, like smartphones and tablets. We therefore report an approach that brings these two lines of research together by using a dynamic motor task that studies hand proximity effects on visual perception.

The question how hand proximity dynamically affects attentional deployment in ongoing movements has recently been addressed in a number of studies (Adam et al., 2012; Festman et al., 2013a,b). For instance, Adam et al. (2012) investigated the effect of hand proximity in a letter discrimination task for both static hand postures and dynamic hand motions below a display. They obtained evidence for superior performance when the hands were close together and thus directly below the letter probe in both settings, suggesting similar mechanisms for static postures and dynamic movements. In contrast, Festman et al. (2013a), who investigated hand proximity effects in a letter discrimination task with continuous hand motions, found performance to be enhanced at far proximity when moving in the direction of the probe (direction effect). The interplay of near-hand and direction effects was examined in a subsequent study by Festman et al. (2013b), in which the right hand moved below the display, while the left hand remained stationary on the side. A direction effect was only present for letter probes on the right side. Movement direction did not influence letter discrimination for left-side probes, suggesting that information from static and dynamically moving hands is integrated into pragmatic maps of attention.

A series of recent studies has provided findings which appear difficult to explain on the basis of attentional mechanisms alone (Reed et al., 2006; Abrams et al., 2008; Davoli et al., 2010; Gozli et al., 2012). For instance, hand proximity does not interact with cue validity, a hallmark of attentional reallocation, in attentional cueing paradigms (Reed et al., 2006; Abrams et al., 2008). Moreover, figure-ground segregation, which is a pre-attentive mechanism, is biased toward perceiving the near-hand object as figure (Cosman and Vecera, 2010). As an alternative to a purely attentional account, Gozli et al. (2012) suggested that near-hand effects also reflect the different involvement of magnocellular (M-cell) and parvocellular (P-cell) neurons in visual processing from near- and far-hand space. M- and P-cells originate in the retina and form two separate pathways up to the LGN, from where they form projections to parietal and temporal regions. M-cells have a high temporal but low spatial resolution due to large receptive fields and fast-conducting axons. They are predominant in the dorsal pathway which is crucial for processing action-related visual information. In contrast, P-cells have a high spatial resolution since they have small receptive fields; they are more prominent in the ventral stream which is specialized in visual object recognition. The association between P- and M-cells and the dorsal and ventral stream is, however, not exclusive, as M-cell contributions in temporal regions are evidenced by research in primates (Ferrera et al., 1994). The modulated visual pathways (MVP) hypothesis (for review, see Taylor et al., 2015) argues that near-hand space is biased toward dorsal M-cell processing whereas far-hand space is biased toward ventral P-cell processing. Consequently, temporal acuity should be enhanced and spatial acuity reduced in near-hand space. In line with their hypothesis, Gozli et al. (2012) found that in near- compared to far-hand space temporal gap detection, where a circle was either presented continuously or with a very small interruption, was enhanced and spatial gap discrimination, i.e., detecting whether a circle was presented with or without a small gap at the top, was reduced.

A recent study by Bush and Vecera (2014) provides an interesting contribution in this context as it points to a potential interaction between parvo- and M-cell engagement and attentional deployment. The authors tested the influence of single vs. bimanual hand proximity on temporal and spatial gap detection. While the findings from Gozli et al. (2012) could be replicated for the comparison of near vs. far bimanual space, the opposite pattern was observed in the single hand conditions. That is, near a single hand temporal sensitivity was impaired and spatial sensitivity was improved compared to opposite from the single hand. These findings suggest that parvo- and M-cell processing are strongly influenced by the span of the attentional window due to the specific hand configuration.

The MVP hypothesis, although conceived as a direct refutation of attention-based accounts, makes clear predictions about changes in visual sensitivity that could otherwise also be accounted for by hypothesizing differential attention deployment. In particular, it provides an interesting and promising theory for hand proximity effects on visual selection but has so far only been tested with static hand postures. Previous studies by Festman et al. (2013a,b) have established that attentional deployment is influenced differently by dynamically moving compared to static hands (direction effect). Therefore, the present study’s goal was to investigate the MVP hypothesis for continuous hand movements. Similar to Gozli et al. (2012), we instructed participants to perform temporal or spatial gap discrimination; their right hand was, however, either moving left- or rightward below the display. During the hand movement lateralized probes appeared contingent upon the right hand passing through one of three positions (left, central, or right). Based on the MVP hypothesis, we predicted improving temporal gap discrimination and impaired spatial gap detection as the hand approached the probe as compared to when the hand moves away from the probe. Furthermore, on the basis of the direction effect of Festman et al. (2013a,b), we predicted better discrimination when moving toward the probe in both tasks.

## Experiment 1: Temporal Gap Discrimination

Experiment 1 tested MVP’s prediction of enhanced temporal perception in near- compared to far-hand space during continuous hand movements. To this end, participants were instructed to detect whether a lateralized ring was presented with or without a short interruption.

### Method

The senior author (MHF) ensured that the study was carried out in accordance with the guidelines of the British Psychological Society (2000), including written informed consent and confidentiality of data as well as personal conduct.

### Participants

A sample of 25 participants aged 20–33 years (*M* = 24; one male), all students at the University of Potsdam with normal or corrected-to-normal vision, participated in the experiment. The sample size was driven by an intention to exceed the sample sizes of our previous published work and was otherwise constrained by convenience sampling. All but two participants reported to be right-handed. The two left-handed participants reported that they nonetheless guide a computer mouse with their right hand. They gave written informed consent and were paid or received course credits for their participation.

### Apparatus

**FIGURE 1:**
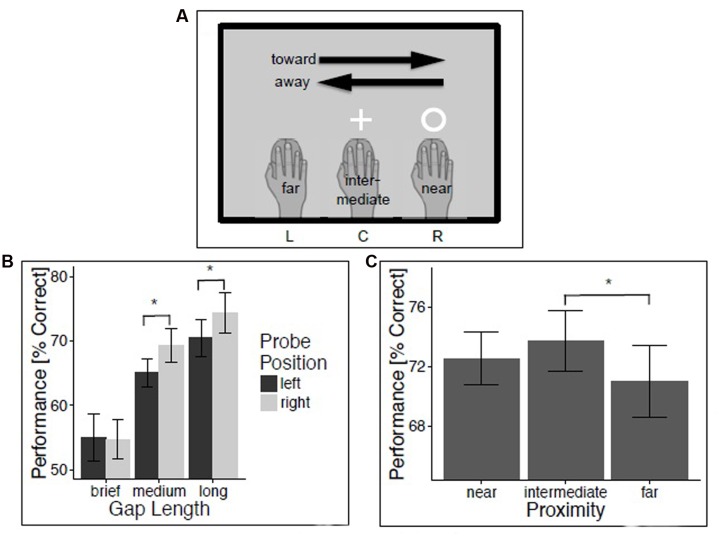
**Experiment 1: Temporal gap discrimination.** Method and results for experiment 1. **(A)** Illustration of trial classification, using a trial with visual probe at location R in the temporal gap discrimination task as an example. **(B)** Temporal gap discrimination performance as a function of probe position and duration. **(C)** Performance as a function of hand proximity. Vertical lines depict 95% confidence intervals.

**Figure 1A** schematically shows the experimental set-up. The experiment was programmed and controlled using Matlab R2012b and Psychophysics Toolbox 3 and was implemented on a Terra computer with Windows 7 (Brainard, 1997). Participants sat in front of a two-layered computer desk. Their right hand was placed on the keyboard shelf below a 22 inch Iiyama ProLite monitor (59 Hz, 1680 × 1050 px) which lay on the desk’s top layer with a tilt angle of 48°. In order to move the monitor’s foot out of the participant’s way, the monitor was physically rotated by 180° while the display was also rotated by 180° (i.e., the monitor was upside-down but the viewing experience was right-side up). Average viewing distance was 52 cm and they were instructed to rest their left hand in their lap. Participants’ view of their hands and arms was blocked by a thin black cape which was attached to the monitor frame during experimental sessions. Hand position was tracked with a wireless high-precision laser mouse (Logitech G700, controlled via Logitech Gaming Software, 200 DPI) that was held with the right hand and allowed hand position–contingent probe onsets. Mouse settings were such that a horizontal (left-to-right) hand movement of 15 cm corresponded to 15 cm cursor movement on the screen; mouse acceleration was disabled. Mouse coordinates—and thus hand positions—were recorded at 60 Hz. As only horizontal movements were task-relevant, vertical movements were disregarded. The mouse was also used for recording participants’ responses. Audio tones were played via headphones.

### Stimuli

Experimental software, raw data and analysis scripts are available via the OSF platform: https://osf.io/pxskj/

All stimuli were displayed in white on a light gray background. A fixation cross (line length: 1°, line thickness: 0.02°) was shown continuously at the screen center to support eye fixation. The visual probe was a ring that was either presented continuously or with a short temporal gap (either 17, 36, or 54 ms) and to the left or right of the fixation cross with 13° eccentricity. Total stimulus duration was identical in the gap and no gap conditions.

### Design

The design consisted of five factors: initial movement/motion direction (two levels: leftward; rightward), critical hand position/probe point (three levels: left = ¼; of horizontal screen width; central = just above the screen center; right = ¾; of horizontal screen width), probe/target position (two levels: left or right of fixation), gap size (three levels: brief, medium, or long), and probe type (two levels: gap or no gap), producing 72 possible combinations per block of randomized trials. There were a total of 10 blocks. Naturally, gap size was zero in the no-gap condition. For analysis, discrimination accuracy (gap vs. no gap) was aggregated along the level combinations of the four remaining factors. That is, per subject, there were 32 different combinations (averaging over probe type); each combination was composed of 20 trials that were aggregated to calculate discrimination accuracy.

### Procedure

At the beginning of a trial, the screen showed a filled semi-circle at the left or right border of the screen to mark the starting position for the hand movement. Participants moved both the cursor on the screen and their hand on the shelf to the indicated side, so that the mouse was positioned under the semi-circle in order to calibrate the set-up for hand-contingent probing. A mouse-click on the semicircle started each trial by presenting an auditory pacing signal: Participants heard two tones, each of 329 Hz and presented with an inter-stimulus interval of 1,200 ms, thereby indicating the desired time from movement start to movement end and thus supporting homogeneous movement times. Only after the playback participants were allowed to start the hand movement; their task was to move the hand from the starting side on the shelf to the other side. During movement execution participants were required to look at the fixation cross; the cursor was not visible to prevent pursuit eye movements. Contingent upon the hand reaching a crucial location along its horizontal trajectory—one of the probe points: left, center, or right—the visual probe appeared for 148 ms in one of two possible locations (left or right of fixation). In half the trials, the visual probe disappeared for the duration of the pre-programmed temporal gap; these gaps were one of three durations (randomized and balanced). In the other half of trials, the visual probe was continuously present. After movement completion, the letters “o” and “c” were presented left and right of fixation in randomized allocation, the letter “o” symbolized a continuously presented ring and the letter “c” a ring with a short interruption. Participants were asked to indicate the perceived probe identity with a mouse click on the “o” or on the “c.” The fixation cross turned green or red for correct or incorrect answers, respectively. Trials were repeated at the end of a block if the movement was initiated prematurely (i.e., before the second audio tone had faded) or if it lasted more than 4 s.

The experiment was completed in one session. The session began with the experimenter introducing the set-up, giving instructions and demonstrating trial execution. Participants then performed practice trials which were excluded from analysis. A checklist ensured that all participants received the same kind of training. The training part of the experiment took typically only a few minutes and participants were allowed to ask questions. Participants then completed the experimental blocks, which took 90–120 min, depending on how liberally participants made use of their discretion to pause and on the number of trials that were repeated.

### Results and Discussion

Per participant trials with movement times below or above two inter-quartile ranges from the median were removed (4.6% of all trials). Average movement time was 1.46 s (*SD* = 282 ms).

### Accuracy Analyses

An initial repeated-measures analysis of variance (ANOVA) on the percentage of correct probe discriminations evaluated the within-subject factors Movement (left and right), Hand Position (left, center, and right), Probe Position (left and right), and Gap Size (brief, medium, and long). We report all effects that were reliable at the conventional *p*-level of 0.05. The analysis revealed a reliable main effect of Gap Size, *F*(2,50) = 49.23, *p* < 0.001, $\eta_{p}^{2}$ = 0.66 (*M* = 58.1, 70.1, and 77.2% for brief, medium, and long temporal gaps, respectively). This effect merely indicates the success of our experimental manipulation of task difficulty. There also was a significant effect of Probe Position, reflecting better performance for probes presented on the right compared to the left side (*M* = 66.2% and *M* = 63.5%, *F*(1,25) = 4.91, *p* < 0.05, $\eta_{p}^{2}$ = 0.16). This probably reflects the fact that the right but not the left hand was active in all conditions. In addition, the effect of Probe Position was modulated by the length of the temporal gap, as indicated by the interaction between Probe Position and Gap Size, *F*(2,50) = 4.34, *p* < 0.05, $\eta_{p}^{2}$ = 0.15. Temporal gap detection was better for right compared to left medium gaps (*M* = 69.3 vs. 65.1%, *t*(25) = -2.44, *p* < 0.05, *d* = 0.48) and right compared to left long gaps (*M* = 74.4 vs. 70.5%, *t*(25) = -2.47, *p* < 0.05, *d* = 0.48) but not for brief temporal gaps, *t*(25) < 1.

In order to evaluate our predictions, trials were then classified with regard to the *proximity* between hand and probe position (*near*: probe and hand position coincide; *intermediate*: hand at central screen position during probe presentation; *far*: probe and hand position at opposite sides of the screen during probe presentation), and with regard to the movement *direction* relative to the probe position (*toward*: probe presented left (or right) and hand moving leftward (or rightward); *away*: probe presented right and hand moving left (and vice versa); **Figure 1A**). A repeated-measures ANOVA evaluated the effects of within-subjects factors Direction (toward and away), Proximity (near, intermediate, and far), and Gap Size (brief, medium, and long) on percentage of correct probe discriminations. Results indicated a strong trend for a significant effect of Proximity, *F*(2,50) = 3.09, *p* = 0.05, $\eta_{p}^{2}$ = 0.11. *T*-tests revealed that this marginal effect of Proximity was driven by a significant difference between intermediate and far proximity trials (65.8 vs. 63.9%, *t*(25) = 2.59, *p* < 0.05, *d* = 0.51). Performance in near proximity trials (64.8%) did not differ from intermediate proximity trials, *t*(25) = -1.27, *p* = 0.22, *d* = 0.25, or far proximity trials, *t*(25) = 1.17, *p* = 0.25, *d* = 0.23.

Since there is evidence that effects of hand proximity are mainly driven by the right hand (Lloyd et al., 2010; Tseng and Bridgeman, 2011), it would be interesting to know if the same pattern of effects can be obtained if the two left handed participants are excluded from the analysis. Therefore, we performed the analyses for right-handed participants only. Consider first the repeated-measures ANOVA with the within-subject factors Movement (left and right), Hand Position (left, center, and right), Probe Position (left and right), and Gap Size (brief, medium, and long). Mirroring the effects from the first analysis, performance was strongly affected by gap size, *F*(2,46) = 40.47, *p* < 0.001, $\eta_{p}^{2}$ = 0.64 (long gaps: *M* = 71.34%, medium gaps: *M* = 66.53%, brief gaps: *M* = 54.44%). Again, accuracy was higher for right-sided (*M* = 65.25%) compared to left-sided temporal probes (*M* = 62.95%), as indicated by the trend for an effect of Probe Position, *F*(1,23) = 3.79, *p* = 0.06, $\eta_{p}^{2}$ = 0.14. There also was an interaction between Probe Position and Gap Size, *F*(2,46) = 3.98, *p* < 0.05, $\eta_{p}^{2}$ = 0.15. Temporal gap detection was better for right compared to left medium gaps (*M* = 64.83 vs. 68.23%, *t*(23) = -2.03, *p* = 0.05, *d* = 0.41) and right compared to left long gaps (*M* = 69.31 vs. 73.37%, *t*(23) = -2.34, *p* < 0.05, *d* = 0.48) but not for brief temporal gaps, *t*(23) < 1. The repeated-measures ANOVA with the within-subjects factors Direction (toward and away), Proximity (near, intermediate, and far), and Gap Size (brief, medium, and long) revealed no significant effect of Proximity, *F*(2,46) = 2.69, *p* = 0.08, $\eta_{p}^{2}$ = 0.10. *T*-tests could, however, confirm the significant difference between intermediate and far proximity trials (63.23 vs. 65.09%, *t*(23) = 2.54, *p* < 0.05, *d* = 0.52). Again, performance in near proximity trials (63.99%) did not differ from intermediate proximity trials, *t*(23) = -1.37, *p* = 0.18, *d* = 0.28, or far proximity trials, *t*(25) < 1.

### Sensitivity and Response Bias Analyses

In order to disentangle effects of the experimental manipulation on sensitivity and response bias in visual detection, we performed an analysis on *d*′ and β values (Macmillan and Creelman, 2005).

The repeated measures ANOVA with within-subject factors Movement (left and right), Hand Position (left, center, and right), Probe Position (left, and right), and Gap Size (brief, medium, and long) on *d*′ revealed that sensitivity was strongly affected by Gap Size, *F*(2,46) = 35.58, *p* < 0.001, $\eta_{p}^{2}$ = 0.61 (long gaps: *M* = 1.49′, medium gaps: *M* = 1.08′, brief gaps: *M* = 0.36′). In addition, there was a strong trend for an interaction between Hand Position, Probe Position, and Gap Size, *F*(4,92) = 2.40, *p* = 0.06, $\eta_{p}^{2}$ = 0.09. In order to specify this interaction, we performed separate repeated-measures ANOVA’s for brief, medium and long gap length trials. While the interaction between Hand Position and Probe Position was not significant for brief temporal gap trials, *F*(2,46) < 1, and long temporal gap trials, *F*(2,46) = 1.22, *p* = 0.31, $\eta_{p}^{2}$ = 0.05, there was a significant interaction for medium temporal gap trials, *F*(2,46) = 4.79, *p* < 0.05, $\eta_{p}^{2}$ = 0.17. Sensitivity was not affected by the position of the probe stimulus when the hand was at the left position, *t*(23) < 1, or right position, *t*(23) = -1.44, *p* = 0.16, *d* = 0.29. When the hand was at the center position, however, sensitivity was higher for right stimuli (*d*′ = 1.18) compared to left stimuli (*d*′ = 0.92), *t*(23) = -2.39, *p* < 0.05, *d* = 0.49.

The repeated-measures ANOVA with within-subject factors Proximity (near, intermediate, and far), Direction (toward and away), and Gap Size (brief, medium, and long) on *d*′ did not show any additional significant effects. The effect of Proximity was not significant, *F*(2,46) = 2.32, *p* = 0.11, $\eta_{p}^{2}$ = 0.09.

The repeated-measures ANOVA with within-subject factors Movement (left and right), Hand Position (left, center, and right), Probe Position (left and right), and Gap Size (brief, medium, and long) on β revealed a significant effect of Gap Size, *F*(2,46) = 5.16, *p* < 0.01, $\eta_{p}^{2}$ = 0.18, suggesting a stronger tendency to respond with “no gap” for briefer temporal gaps (brief gaps: *M* = 2.20, medium gaps: *M* = 1.82, long gaps: *M* = 1.27). There also was a trend for an interaction between Gap Size and Probe Position, *F*(2,46) = 2.93, *p* = 0.06, $\eta_{p}^{2}$ = 0.11. The tendency to respond with “no gap” was stronger for right stimuli (β = 2.34) compared to left stimuli (β = 1.30) only in medium temporal gap trials, *t*(23) = -2.46, *p* < 0.05, *d* = 0.50, but not in brief temporal gap trials, *t*(23) < 1, or long temporal gap trials, *t*(23) < 1. Moreover, there was a significant interaction between Hand Position, Probe Position, and Gap Size, *F*(4,92) = 2.74, *p* < 0.05, $\eta_{p}^{2}$ = 0.11. In order to understand this three-way interaction, we performed separate repeated-measures ANOVA’s for brief, medium and long temporal gap trials. While the interaction between Hand Position and Probe Position was not significant for medium temporal gap trials, *F*(2,46) < 1, and long temporal gap trials, *F*(2,46) = 1.13, *p* = 0.33, $\eta_{p}^{2}$ = 0.05, there was a significant interaction for brief temporal gap trials, *F*(2,46) = 3.48, *p* < 0.05, $\eta_{p}^{2}$ = 0.13. *T*-tests, however, revealed no significant difference in response bias between left- and right-side stimuli for left hand position trials, *t*(23) = -1.78, *p* = 0.09, *d* = 0.36, center hand position trials, *t*(23) = 1.72, *p* = 0.10, *d* = 0.35, or right hand position trials, *t*(23) < 1.

The repeated-measures ANOVA with within-subject factors Proximity (near, intermediate, and far), Direction (toward and away), and Gap Size (brief, medium, and long) on β did not show any additional significant effects. The effect of Proximity was not significant, *F*(2,46) = 2.65, *p* = 0.08, $\eta_{p}^{2}$ = 0.10.

Overall, Experiment 1 demonstrated a successful manipulation of temporal gap discrimination difficulty and a selective modulation of performance by attentional factors. Specifically, it revealed a hand proximity effect in (partial) accordance with MVP but no further modulation of hand proximity by movement direction. Sensitivity and bias were consistently affected by our gap duration manipulation; the slightly better sensitivity for right compared to left stimuli when the hand was centered probably reflects the hand dominance of our participants. We now turn to an analysis of spatial gap discrimination performance in the same paradigm.

## Experiment 2: Spatial Gap Discrimination

In Experiment 2, we tested MVP’s prediction that spatial perception should be superior in far compared to near-hand space during continuous hand movements. Consequently, participants were instructed to detect whether a lateralized ring was presented with or without a small gap.

### Method

The senior author (MHF) ensured that the study was carried out in accordance with the guidelines of the British Psychological Society (2000), including written informed consent and confidentiality of data as well as personal conduct.

### Participants

A new sample of 23 participants aged 20–33 years (mean age = 24; one male), all students at the University of Potsdam with normal or corrected-to-normal vision, participated in the experiment. The sample size was driven by an intention to exceed the sample sizes of our previous published work and was otherwise constrained by convenience sampling. All but two participants reported to be right-handed. The two left-handed participants reported that they nonetheless guide a computer mouse with their right hand. They gave written informed consent and were paid or received course credits for their participation.

### Apparatus

The apparatus and software were identical to Experiment 1.

### Stimuli

All stimuli were displayed in white on a black background. The change of background from gray to black was the result of a compromise between maintaining reasonably high performance and keeping spatial parameters comparable. A fixation cross (line length: 1°, line thickness: 0.02°) was shown continuously at the screen center to support eye fixation. The visual probe was either a Landolt-C–like ring or a closed ring of 1.24°of visual angle. The gap of the Landolt-C–like probe was 0.25° or 0.19° or 0.12°, based on the average viewing distance of 52 cm. It appeared to the left or right of the fixation cross with 13° eccentricity.

### Design

The design was identical to Experiment 1.

### Procedure

Apart from the fact that the gap in the visual probe was spatial and not temporal the procedure was identical to Experiment 1. In half the trials, the probe was a ring which was presented with a gap. Gaps were one of three sizes (randomized and balanced) and the gap location was always at the top of the ring (at the 12 o’clock position, as in Gozli et al., 2012). In the other half of trials, the visual probe was a ring without gap. As in Experiment 1, the letters “o” and “c” were presented left and right of fixation in randomized allocation after movement completion for participants to report their perception of the probe per mouse click. Here, the letter “o” symbolized a closed ring and the letter “c” an open one.

### Results and Discussion

Per participant trials with movement times below or above two inter-quartile ranges from the median were removed (4.6% of all trials). Average movement time was 1.66 s (*SD* = 293 ms).

### Accuracy Analyses

**FIGURE 2:**
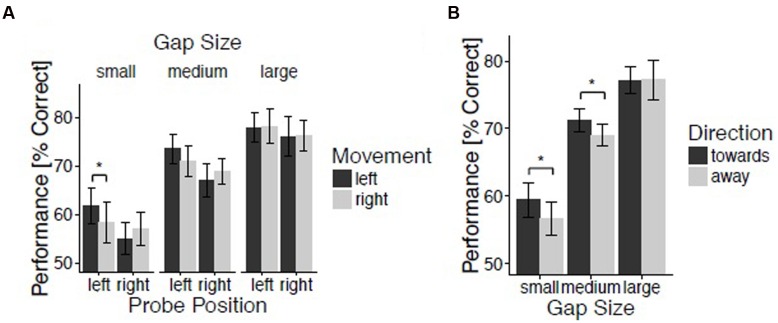
**Spatial gap discrimination.** Results for experiment 2. **(A)** Spatial gap discrimination performance as a function of movement, probe position, and gap size. **(B)** Performance as a function of direction and gap size. Vertical lines reflect 95% confidence intervals.

The percentages of correctly identified probes were submitted to an initial repeated-measures ANOVA evaluating the within-subject factors Movement (left and right), Hand Position (left, center, and right), Probe Position (left and right), and Gap Size (small, medium, and large). The analysis revealed a significant main effect of Gap Size, *F*(2,48) = 114.70, *p* < 0.001, $\eta_{p}^{2}$ = 0.83, reflecting improving performance for increasing spatial gap sizes (*M* = 58.1, 70.1, and 77.2% for small, medium, and large gaps, respectively). This confirms the success of our intended manipulation of task difficulty. There was a marginally reliable three-way interaction between Gap Size, Probe Position, and Movement, *F*(2,48) = 2.80, *p* = 0.07, $\eta_{p}^{2}$ = 0.10 (**Figure 2A**). In order to understand this three-way interaction, repeated-measures ANOVA’s with the factors Movement and Probe Position were performed separately for small, medium, and large spatial gap trials. For small gap trials, the interaction between Movement and Probe Position showed a strong trend toward significance, indicating the presence of a direction effect, *F*(1,24) = 3.58, *p* = 0.07, $\eta_{p}^{2}$ = 0.13. Indeed, spatial gap detection was enhanced for probes presented on the left side when moving leftward (*M* = 61.8%) as compared to when moving rightward (*M* = 58.3%; *t*(24) = 1.81, *p* < 0.05, *d* = 0.36 (one-tailed). The effect of Movement for probes presented on the right side was not significant, *t*(24) = -1.07, *p* = 0.07 (one-tailed), *d* = 0.21. For both medium and large gap trials the interaction between Movement and Probe Position was not significant, *F*(1,24) = 3.21, *p* = 0.09, $\eta_{p}^{2}$ = 0.12, and *F*(1,24) < 1, respectively.

Trials were again classified with regard to the *proximity* between hand and probe position, and with regard to movement *direction* relative to probe position (as described above). A repeated-measures ANOVA with the within-subjects factors Direction (toward and away), Proximity (near, intermediate, and far), and Gap Size (small, medium, and large) revealed a trend for an interaction between Direction and Gap Size, *F*(2,48) = 2.80, *p* = 0.07, $\eta_{p}^{2}$ = 0.10. In order to understand this marginal interaction, *t*-tests comparing trials with movements directed toward and away from the probe were performed for small, medium, and large gap trials. The effect of Direction was significant for small gap trials, *t*(24) = 1.89, *p* < 0.05, *d* = 0.38 (one-tailed), and medium gap trials, *t*(24) = 1.79, *p* < 0.05, *d* = 0.36 (one-tailed), but not for large gap trials, *t*(24) < 1, (**Figure 2B**). For small gap trials, discrimination was better when moving toward [*M* = 59.5% (small gap) and *M* = 71.3% (medium gap)] as opposed to away from the probe [*M* = 56.7% (small gap) and *M* = 69.0% (medium gap)], thereby reproducing the effect of movement direction previously reported by Festman et al. (2013a,b).

Again, we performed both analyses for right handed participants alone. The repeated-measures ANOVA with the within-subject factors Movement (left and right), Hand Position (left, center, and right), Probe Position (left and right), and Gap Size (brief, medium, and long) revealed an effect of Gap Size, *F*(2,44) = 105.95, *p* < 0.001, $\eta_{p}^{2}$ = 0.83 (large gaps: *M* = 76,92%, medium gaps: *M* = 70.43%, and brief gaps: *M* = 58.44%). The three-way interaction between Gap Size, Probe Position, and Movement was, however, not significant, *F*(2,44) = 1.90, *p* = 0.16, $\eta_{p}^{2}$ = 0.08.

The repeated-measures ANOVA with the within-subjects factors Direction (toward and away), Proximity (near, intermediate, and far), and Gap Size (brief, medium, and long) on accuracy in right-handed participants did not replicate the interaction between Direction and Gap Size, *F*(2,44) = 1.90, *p* = 0.16, $\eta_{p}^{2}$ = 0.08.

### Sensitivity and Response Bias Analyses

The repeated-measures ANOVA with within-subject factors Movement (left and right), Hand Position (left, center, and right), Probe Position (left and right), and Gap Size (small, medium, and large) on *d*′ revealed a strong effect of Gap Size, *F*(2,46) = 74.68, *p* < 0.001, $\eta_{p}^{2}$ = 0.76 (large gaps: *M* = 1.94, medium gaps: *M* = 1.35, and small gaps: *M* = 0.60). In addition, there was a significant interaction between Movement, Probe Position, and Gap Size, *F*(4,92) = 5.26, *p* = 0.06, $\eta_{p}^{2}$ = 0.19. In order to specify this interaction, we performed separate repeated-measures ANOVAs for small, medium, and large gap trials. While the interaction between Hand Position and Probe Position was not significant for large gap trials, *F*(1,23) < 1, there was a significant interaction for small spatial gap trials, *F*(1,23) = 6.18, *p* < 0.05, $\eta_{p}^{2}$ = 0.21, and for medium spatial gap trials, *F*(1,23) = 4.14, *p* < 0.05, $\eta_{p}^{2}$ = 0.15. For small gap trials, when moving leftward, sensitivity was higher for left-sided stimuli (*d*′ = 0.89) compared to right-sided stimuli (*d*′ = 0.34), *t*(23) = 2.86, *p* < 0.01, *d* = 0.58. When moving rightward, sensitivity was not affected by the stimulus position, *t*(23) < 1. Similarly, for medium gap trials, when moving left, sensitivity was higher for left-sided stimuli (*d*′ = 1.65) compared to right-sided stimuli (*d*′ = 1.07), *t*(23) = 2.99, *p* < 0.01, *d* = 0.61. When moving rightward, sensitivity was not affected by the stimulus position, *t*(23) = 1.03, *p* = 0.31, *d* = 0.21.

The repeated-measures ANOVA with within-subject factors Proximity (near, intermediate, and far), Direction (toward and away), and Gap Size (small, medium, and large) on *d*′ revealed a significant interaction between Direction and Gap Size. For small gap trials, sensitivity was enhanced when moving toward (*d*′ = 0.73) compared to away from the stimulus (*d*′ = 0.46), *t*(23) = -2.49, *p* < 0.05, *d* = 0.26. Similarly, for medium gap trials, sensitivity was higher when moving toward (*d*′ = 1.45) compared to moving away (*d*′ = 1.25) from the stimulus, *t*(23) = -2.03, *p* = 0.05, *d* = 0.20. Movement direction did not affect sensitivity in large gap trials, *t*(23) < 1.

The repeated-measures ANOVA with within-subject factors Movement (left and right), Hand Position (left, center, and right), Probe Position (left and right), and Gap Size (small, medium, and large) on β revealed a significant effect of Gap Size, *F*(2,46) = 4.89, *p* < 0.05, $\eta_{p}^{2}$ = 0.18, due to a stronger tendency to respond with “no gap” for small and medium compared to large spatial gaps (small gaps: *M* = 2.27, medium gaps: *M* = 2.53, and large gaps: *M* = 1.68). All other effects were not significant, all *p* > = 0.11.

The repeated measures ANOVA with within-subject factors Proximity (near, intermediate, and far), Direction (toward and away), and Gap Size (small, medium, and large) on β revealed no additional significant effects, all *p* > = 0.17.

Experiment 2 successfully manipulated spatial gap discrimination difficulty and obtained two relevant findings. First, we observed no modulation of performance by proximity; this result conflicts with the prediction derived from the MVP hypothesis. Secondly, there was a direction effect, that is, enhanced visual discrimination when moving toward as compared to away from a visual probe. Sensitivity and bias behaved again consistently across conditions. This time, for small and medium spatial gaps, there were congruity effects in sensitivity for leftward movements and for movements toward the visual probes. We will discuss implications of these findings and of the results from temporal gap discrimination below.

## General Discussion

The current study was inspired by the fact that visual attention deployment and movement planning seem to be closely coupled. However, previous work appears to be divided into two strands which, we have briefly reviewed in our Introduction: One line of research has established enhanced visual target perception when the hand moves toward the intended target object (a direction effect). The other line of research has reported reduced visual efficiency as a result of higher attention allocation near the resting hands (a near-hand effect). The apparent conflict between these two outcomes could reflect the comparison of moving vs. resting postures. Thus, we set out to simultaneously assess effects of both movement direction and hand proximity on visual discrimination.

Our aim was to test the MVP hypothesis (Gozli et al., 2012; Goodhew et al., 2015; Taylor et al., 2015), according to which a combination of attentional and neurophysiological factors contribute to the observed interactions between perception and action, as was briefly reviewed in the Introduction. A specific prediction of the MVP hypothesis is an enhancement of temporal and an impairment of spatial acuity in near-hand compared to far-hand space. Following Gozli et al. (2012), we measured visual discrimination performance in both a temporal task (Experiment 1) and a spatial task (Experiment 2) while observers performed dynamic hand movements toward or away from visual probes that were presented contingent upon their hands’ position.

Our analyses revealed evidence for an effect of hand-target proximity in temporal but not in spatial gap discrimination. In addition, we obtained (weak) statistical evidence for a directionality effect, that is, enhanced performance when moving toward compared to away from the probe, but only in spatial gap discrimination. How does this mixed outcome qualify the MVP hypothesis? And what are its implications for our understanding of the relationship between attention deployment and action more generally? We will address these questions in turn.

The MVP hypothesis predicts a trade-off between temporal vs. spatial perceptual abilities in near compared to far-hand space on the basis of a bias toward M-cell vs. P-cell involvement. Our finding of enhanced temporal gap discrimination at intermediate rather than near proximity, together with the complete absence of an effect of hand-target proximity on spatial gap discrimination, reflects a failure to replicate Gozli et al. (2012), and is therefore clearly in conflict with this idea. This outcome cannot be attributed to a lack of strength of our manipulation of task difficulty, given that there was a clear effect of gap size in both experiments. Moreover, the absence of a proximity effect in the spatial discrimination task can also not be attributed to a lack of attentional involvement because, we found better spatial probe discrimination when the hand was moving toward small or medium-sized probes, thus reflecting an attentional benefit in perceptually difficult conditions, consistent with the direction effect previously reported by Festman et al. (2013a,b).

It is, however, important to realize that the larger probe eccentricities in the present method, namely 13 degrees compared to only 4 degrees in the study by Gozli et al. (2012), may have contributed to the difference in outcome. The larger eccentricities here were a necessary implication of the active hand movement manipulation, we wished to introduce but this design decision also limits the comparability with the original study. While probe discriminability generally drops with increasing eccentricity, we are unaware of any published studies of eccentricity effects on the relative contributions of the two pathways to probe discrimination (but see Livingstone and Hubel, 1988 and Dacey and Petersen, 1992, for evidence from tracer injection). This important issue needs to be addressed by further investigations.

In this context, it is informative to look at the methodological details of previous research on proximity effects for moving hands (Adam et al., 2012; Festman et al., 2013a,b): All previous studies used letter discrimination as a task to examine visual selection performance, which – according to the MVP hypothesis – should also be enhanced in far-hand space, due to its higher demands on spatial compared to temporal processing. Interestingly, none of these studies found an enhancement of letter discrimination performance in far-hand space. While in Festman et al. (2013a,b) performance was not affected by hand proximity as such, Adam et al. (2012) observed better performance in near-hand space. At this point, we can only speculate why a near-hand effect does not seem to occur in spatial tasks when the hands are moving. It might be envisioned that a temporal/spatial processing biases for far/near-hand space was overshadowed by the engagement of pragmatic maps of attention, due to active movement of the hands (cf. Rizzolatti et al., 1987, 1994). In line with this idea, in the spatial gap discrimination task, where an involvement of pragmatic attentional maps was indicated by the modulatory effect of movement direction, an effect of hand-target proximity was entirely absent.

In the same regard, another interesting finding of the current study is that, although statistically the intermediate and near conditions were equivalent, visual selection was numerically best at intermediate proximity in the temporal gap task. Festman et al. (2013b) have previously shown that stationary and dynamically moving hands combine in their effect on visual selection. Since participants in the present study were instructed to rest their left hand in their lap, the two hands of a participant were closest together whenever the right hand reached the central position under the display, which reflects our intermediate hand proximity condition. As a consequence, there was a partial overlap of pragmatic maps from the left and the right hands, which might explain enhanced performance at intermediate proximity. The absence of this same overlap effect during spatial gap discrimination would be consistent with MVP, according to which an overlap of hand-related processing areas is detrimental in spatial tasks.

We observed evidence for a directionality effect in the spatial gap discrimination task, as reflected in enhanced performance when moving toward as compared to moving away from the probe, which provides a replication of the previously established directionality effects in spatial tasks (Festman et al., 2013a,b). This finding is in line with the idea that attention is shifted ahead of the current hand position toward action-relevant locations and thereby extends earlier findings suggesting that attentional allocation is not only influenced by stationary hand positions but by continuous manual motion (e.g., Tipper et al., 1992; Fischer, 1997).

Recent research suggests that hand configurations, that is, uni- vs. bimanual postures, modulate parvo and M-cell contributions by altering the window of attention (Bush and Vecera, 2014). Bimanual postures lead to a spread of attention across the whole display favoring M-cell processing. Unimanual postures, in contrast, create a small focused area close to the hand inducing a bias toward P-cell engagement. Our findings point to manual motion as another variable which further complicates the underlying mechanism. Such an interplay is indicated by the presence of an effect of movement direction and the concurrent absence of an influence of hand proximity in the spatial gap task.

Our findings are also in line with a bimodal neuronal integration mechanism that combines both visual and tactile information from the body (Graziano and Gross, 1998). This, in turn, provides an online, multisensory representation of visual information in peripersonal space centered on active body parts (see Graziano and Gross, 1998; Graziano, 2001) and is also involved in directing spatial attention (Bremmer et al., 2001; Halligan et al., 2003). This bimodal integration mechanism has been made responsible for earlier findings of a near-hand advantage for visual attention in visual search, detection, and attentional blink tasks (cf. Abrams et al., 2008), and has also been proposed to account for the modulating effects of hand position in flanker interference tasks (Davoli and Brockmole, 2012).

The present study, and other related findings emerging from recent research on the effects of hand proximity on visual processing, have implications for how, we best interact with touch devices in everyday activities. Clearly, our attention allocation abilities and the resulting extraction of information from the visual display can be affected by both the stationary position and the ongoing movements of our hands. The specific contributions of the two factors will depend on whether, we aim to extract temporal or spatial information from the display: While temporal information is liable to proximity effects, spatial information seems to be more sensitive to direction effects that are triggered by ongoing manual activity. A striking example in this context is the demonstration that hand position can even affect very fundamental processes of word perception, such as the Stroop-effect (Davoli et al., 2010). That is, the well-established interference from color words in a color naming task was abolished in near hand space. The optimization of touch device applications in terms of spatial and temporal integration of finger gestures and visual presentations on the display can certainly benefit from the new insights provided by this field of research.

## Ethics Statement

The study was conducted in accordance with the ethical standards laid out in the Declaration of Helsinki. Participants gave written informed consent.

## Author Contributions

The experiments were conceived by MF, data were collected and analyzed by MW, the report was written by MW and MF.

## Conflict of Interest Statement

The authors declare that the research was conducted in the absence of any commercial or financial relationships that could be construed as a potential conflict of interest.
